# Study on the Performance and Solidification Mechanism of Multi-Source Solid-Waste-Based Soft Soil Solidification Materials

**DOI:** 10.3390/ma16134517

**Published:** 2023-06-21

**Authors:** Keyi Qiu, Guodong Zeng, Benan Shu, Dongmei Luo

**Affiliations:** 1 School of Civil Engineering, Foshan University, Foshan 528000, China; qiuky1998@163.com (K.Q.); dmluo@fosu.edu.cn (D.L.); 2 Foshan Transportation Science and Technology Co., Ltd., Foshan 528000, China; 2112161023@stu.fosu.edu.cn

**Keywords:** soft soil, solidification mechanism, solid waste, unconfined compressive strength

## Abstract

In this paper, ground granulated blast furnace slag, steel slag, red mud, waste ceramic powder, and desulfurization gypsum were used as raw materials to develop a kind of multi-source solid-waste-based soft soil solidification material. Three ratios and the strength activity index were used to determine the fractions of different solid wastes. The mineralogical and microstructural characterization was analyzed by X-ray diffraction (XRD), scanning electron microscope (SEM), and thermogravimetric analysis–differential scanning calorimetry (TG&DSC) tests. The results showed that the unconfined compressive strength of the three types of soft soil increases with an increase in the content of the solidifying agent. The failure strain of the stabilized soil decreases from 1.0–1.3% to 0.75–1.0%, and the failure mode gradually changes from plastic failure to brittle failure. The optimum content of the solidifying agent was determined to be 17% (the lime saturation factor (KH), silica modulus (SM), and alumina modulus (IM) of the solidifying agent were set to 0.68, 1.74, and 1.70, respectively), and the unconfined compressive strength (28 d) of the solidified soil (sandy soil, silty clay, and organic clay) was 3.16 MPa, 2.05 MPa, 1.04 MPa, respectively. Both measurements can satisfy the technical requirements for a cement–soil mixing pile, suggesting the possibility of using various types of solid waste as a substitute for cement.

## 1. Introduction

Industrial byproducts such as mineral separation, power supply, and metal smelting are showing a continuous growth trend. However, the treatment of industrial waste has become a major challenge in the global environmental and energy field, with a utilization rate of less than 60% [1]. The general industrial solid waste generated in China alone has risen from 3.68 billion tons in 2020 to 3.97 billion tons in 2021, with a comprehensive utilization rate of around 55% [2]. With the new development philosophy of carbon peaking and carbon neutrality goals from the government of China, a consensus has been reached to take the green, low-carbon, and high-quality development path. Compared with the thermal decomposition and sanitary landfill of solid waste, the efficient reuse of solid waste has become a mainstream trend [3,4].

Cement is widely used in the solidification of soft soil via simple in situ mixing or grouting due to its mature manufacturing process, excellent stability, and simple operation [5,6]. However, the global cement industry accounts for 9–10% of the world’s anthropogenic carbon dioxide emissions [7], and cement is considered a non-renewable energy source that has harmful effects on air quality and climate change [8].

Currently, increasing attention has been paid to the use of industrial or agricultural byproducts to replace part or all of the cement for soft soil solidification. Compared with cement production, solid-waste-based solidification materials can achieve a reduction in carbon dioxide emissions of 60% to 80% [9,10]. Goodarzi et al. [11] found that ground granulated blast furnace slag (GGBS) can be used to solidify zinc-contaminated clay. Compared with cement, MgO-GGBS stabilized mixtures showed a better Zn immobilization efficiency. Francisca et al. [12] investigate the effect of steel slag (SS) addition as a stabilizer in soil. It can be observed that the addition of SS in compacted loess specimens improves mechanical behavior measured by the UCS test. The UCS of 12% SS-loess specimens increased by 200% after 56 days of curing. Mohit et al. [13] studied the properties of samples made using ceramic powder and limestone powder as a substitute for cement and evaluated the mechanical properties and durability of the mortar samples. It was found that the mortar samples with 20% ceramic powder and 15% limestone powder formed calcium aluminum silicate hydrate gel by consuming calcium hydroxide, resulting in improved strength and durability. Wang et al. [14] used ground granulated blast furnace slag and desulfurization gypsum as the main material for soft soil solidification. Compared with cement-solidified soil, the new stabilizer can effectively improve the water stability and strength of the soil. The higher strength of the solidified soil was mainly attributed to the pore filling effect of ettringite.

However, most research has focused on one or two types of solid waste that partially replace traditional solidifying agents such as cement or lime [11,12,15,16], and there are few studies about the use of three or more solid wastes for soil stabilization. Recently, the three ratios and strength activity index originally developed for cement clinker have been successfully applied to the stabilization of soil based on multi-source solid waste. Wu et al. [17] investigated the modification potential of steel slag on expansive soils. As a result, the mechanical properties of the steel slag-based composite binders were significantly improved by adjusting the composition in three ratios. These improved soils were sturdy enough to be used as a sub-base for highway pavement.

In this paper, a kind of multi-source solid-waste-based soft soil solidification material was developed by using solid waste from Foshan as raw materials, including ground granulated blast furnace slag, steel slag, red mud, waste ceramic powder, and desulfurization gypsum, and the concepts of three ratios and the strength activity index originally developed for the cement clinker were used to determine the fractions of different solid wastes. The goal of this research was to characterize the solidified soil by artificially preparing the solidifying agent and to reveal the solidification mechanism.

## 2. Materials and Methods

### 2.1. Three Ratios

During the process of cement production, controlling the proportion between various oxides in cement production is more reflective of the effect on clinker mineral composition and properties than controlling the content of each oxide separately. However, the actual reactive or active components are not individual oxides. For example, dicalcium silicate (CaSiO_4_) or tricalcium silicate (Ca_3_SiO_5_) is the main active component in ordinary Portland cement, but traditional elemental analysis reports its chemical composition to be oxide components. Three ratios that indicate the relative content of different oxides are used as indicators for the production of ordinary Portland cement, including lime saturation factor (KH), silica modulus (SM), and alumina modulus (IM) [18,19].

By introducing three ratios to optimize the proportions of CaO, SiO_2_, Al_2_O_3_, and Fe_2_O_3_ in the system, the fraction of each solid waste can be determined. Three ratios can be determined by Equations (1)–(3) [20,21]:

(1) $$\mathrm{KH}=\frac{CaO-1.65\times Al_{2}O_{3}-0.35\times Fe_{2}O_{3}}{2.8\times SiO_{2}}$$

(2) $$SM=\frac{SiO_{2}}{Al_{2}O_{3}+Fe_{2}O_{3}}$$

(3) $$IM=\frac{Al_{2}O_{3}}{Fe_{2}O_{3}}$$

where CaO, SiO_2_, Al_2_O_3_, and Fe_2_O_3_ represent the mass-based fractions of the solid waste. The ranges of KH, SM, and IM in cement clinker are 0.67–1.0, 1.7–2.7, and 0.9–1.7, respectively [21,22].

Not all components in solid waste participate in reactions; some may only play a physical filling role. To reduce the impact of the inert fraction, the strength activity index (SAI) is used to evaluate active oxides [23]. The SAI of solid waste is defined as [24]

(4) $$SAI=\frac{A}{B}\times 100\%$$

where A is the unconfined compressive strength of standard size mortar cubes of 40 × 40 × 40 mm prepared with 70 wt% reference cement plus 30 wt% solid waste, and B is the counterpart made from reference cement that is only at a water–binder ratio of 0.5 cured under the same conditions for 28 days.

According to the best practices and implementation strategies recommended by Wu et al. [25], the design scheme of multi-source solid-waste-based soft soil solidification materials consists of the following four steps: (1) drying and grinding the solid waste to a fineness similar to cement (sieve residue of square hole with 80 µm ≤10% [26]); (2) determining the oxide fractions (i.e., CaO, SiO_2_, Al_2_O_3_, and Fe_2_O_3_) of the solid waste by elemental analysis such as X-ray fluorescence; (3) testing the strength activity index of the solid waste by Equation (4); (4) combining the three ratios to determine the proportion of solid waste. The process framework is summarized in Table 1.

### 2.2. Materials

**Figure 1:**
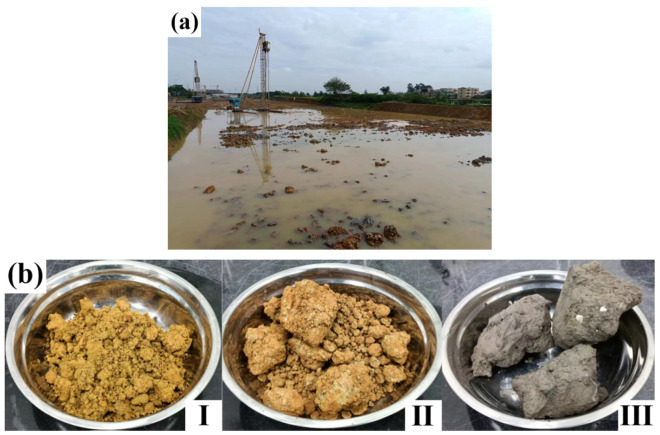
The studied soil sample. (**a**) Sampling location. (**b**) Appearance of the studied soil sample: (**Ⅰ**) silty sand; (**Ⅱ**) silty clay; (**Ⅲ**) organic clay.

The studied soil was sampled from Foshan, a city in southeast China, at depths greater than 3.0 m below the surface (as shown in Figure 1a,b). The soil sample was first dried at 50–60 °C and then ground and screened using a 2 mm sieve to obtain the experimental sample [27]. Geotechnical tests were carried out, and the results of the basic parameters are shown in Table 2, which were determined based on the pertinent American Society for Testing and Materials (ASTM) standard methods. According to the Unified Soil Classification System specified by ASTM D2487 [28], test soils Ⅰ, Ⅱ, and Ⅲ were classified as silty sand (SM), silty sand (SM), and organic clay (OH), respectively. The organic matter contents of test soils Ⅰ, Ⅱ, and Ⅲ were 0%, 0%, and 5.31%, respectively, according to ASTM D2974 [29]. The particle size distribution (clay, silt, and sand fractions) was determined using the hydrometer method as per ASTM D7928 [30].

**Figure 2:**
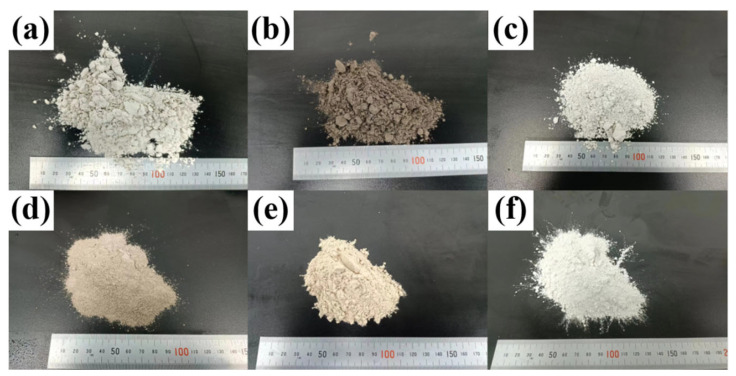
Multi-source solid waste: (**a**) ground granulated blast furnace slag, (**b**) steel slag, (**c**) waste ceramic powder, (**d**) red mud, (**e**) desulfurization gypsum, and (**f**) lime.

Foshan has abundant solid waste, including ground granulated blast furnace slag, steel slag, waste ceramic powder, red mud, and desulfurization gypsum (Figure 2). The ground granulated blast furnace slag and steel slag, which are byproducts of iron production in blast furnaces, were purchased from Foshan Baogang Steel Group Co., Ltd. (Foshan, China). The waste ceramic powder was collected from the waste dust generated in the ceramic processing production line of Foshan Xinghui Ceramics Co., Ltd. (Foshan, China). The red mud, which is a kind of strong alkaline solid waste produced by alumina production from refined bauxite, was obtained from Foshan Sanshui Aluminum Industry Co., Ltd. (Foshan, China). The desulfurization gypsum is an industrial byproduct produced by limestone–gypsum wet desulfurization to treat SO_2_ produced by Foshan Sanshui Hengyi Thermal Power Co., Ltd. (Foshan, China). The chemical compositions of the solid wastes were studied by an X-ray fluorescence (XRF) spectrometer (PANalytical PW5400, Almelo, The Netherlands). The spectral range is 350 nm–2500 nm. The sampling interval was 3 nm (350 nm–1000 nm) and 10 nm (1000 nm–2500 nm), and the sampling resolution of the spectra was 1 nm. All the solid wastes were air-dried at room temperature, crushed, and passed through a 0.075 mm mesh sieve before XRF spectrum measurement [31]. Table 3 lists the oxide composition and loss on ignition values for raw materials [32]. Table 4 shows the SAI of the solid waste. Moreover, industrial-grade lime was chosen to provide additional CaO. The PO 42.5 Portland cement was purchased from Taiwan Cement Corporation Co., Ltd. (Taiwan, China).

### 2.3. Sample Preparation

The three ratios of cement clinker (KH = 0.67–1.0; SM = 1.7–2.7; IM = 0.9–1.7) were used as reference to design different solid waste components (KH, SM, and IM of the solidifying agent were set to 0.68, 1.74, and 1.70, respectively). A total of 60% of the cement is substituted with the solid waste mixture. The total amount of the solidifying agent incorporated is 14%, 17%, and 20% of the dry soil mass, and the water to binder ratio is set to 0.7. The solidifying agent consisted of 24, 18, 12, 4, 22, and 20 wt% of ground granulated blast furnace slag, steel slag, waste ceramic powder, red mud, lime, and desulfurization gypsum, respectively.

**Figure 3:**
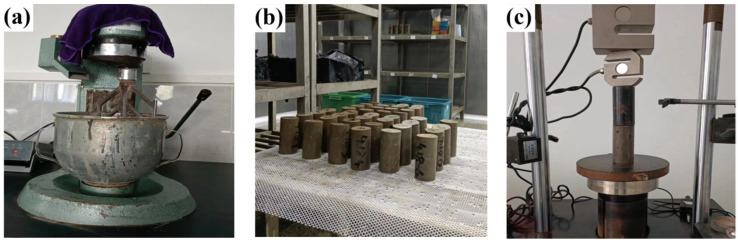
Process of the laboratory experiment of the solidified soil: (**a**) stirring, (**b**) curing and (**c**) unconfined compressive strength test.

The stirring, forming, curing, and strength tests of solidified soil are referred to in the specification of “Mix Proportion Design Of Cement Soil” (JGJ/T 233–2011, Beijing, China), which includes the following three steps: (1) Pour the weighed water into the mixing pot, add the solid-waste-based binders, and mix quickly with the soil mixing knife. Then, add the dry soil and place it in the cement paste mixer for mixing, setting the mixing speed of the apparatus to 2 min at 300 rpm and then 2 min at 1000 rpm (Figure 3a). (2) The mixed slurry is poured into a mold that is 50 mm in diameter and 100 mm in height. The surface is leveled with a trowel, placed on a vibrating table for 2 min to vibrate, and then covered with plastic wrap. (3) After 48 h of demolding, the solidified soil samples were placed in the standard curing room with a relative humidity of 95% and a temperature of 20 ± 5 °C (Figure 3b). The unconfined compressive strength (UCS) of the solidified soil is tested on the machine (Yuchuang Fluid Technology Co., Ltd., Zhangjiagang, China), and the loading rate of force is set to 80 N/s (Figure 3c).

### 2.4. Mineralogical and Microstructural Analyses

Fragments from the failed specimens of unconfined compression testing were lyophilized using the following procedures: immerse in liquid nitrogen at −196 °C for instant freezing and then transfer into a SZFD-20A freeze-dryer (Shanghai Shunzhi Instrument Corp., Shanghai, China) for sublimation for approximately 24 h [33]. The dried specimen was then analyzed by XRD, SEM, and TGA.

#### 2.4.1. X-ray Diffraction (XRD)

XRD test was operated on the machine (Rigaku SmartLab SE intelligent X-ray diffractometer, Tokyo, Japan). The instrument uses Cu-Kα radiation with a generator power of 3 kilowatts, an operating voltage of 40 kV, and a current of 20 mA. The scanning angle ranges from 5° to 70°, and the scanning speed is 2°/min. Freeze-dried powder samples with particles of <75 μm in size were prepared from stabilized soil samples. Computer software (MDI Jade 6) was used to determine the identity of the mineralogical compositions of the specimen by comparison to a database, such as the ICDD PDF-4+ [34].

#### 2.4.2. Scanning Electron Microscope (SEM)

SEM test was performed using the Zeiss Merlin high-resolution field emission scanning electron microscope, Germany. The surface of the freeze-dried samples was coated with a layer of gold with a thickness of 200 Å–300 Å (1Å = 0.1 nm) to provide conductivity and prevent the accumulation of charges on the surface [35]. The accelerating voltage ranged from 0.05 kV to 30 kV, and magnification could be adjusted from 1000× to 3000×.

#### 2.4.3. Thermogravimetric Analysis (TGA)

TGA test was performed using the TG 209 F1 Libra Thermogravimetric Analyzer NETZSCH, Germany. Freeze-dried powder samples with particles of <75 μm in size were prepared from stabilized soil samples. The test was conducted under nitrogen environment at a heating rate of 10 °C/min, with the temperature range set between 20 to 800 °C.

## 3. Results and Discussion

### 3.1. Analysis of UCS Test Results

#### 3.1.1. UCS

**Figure 4:**
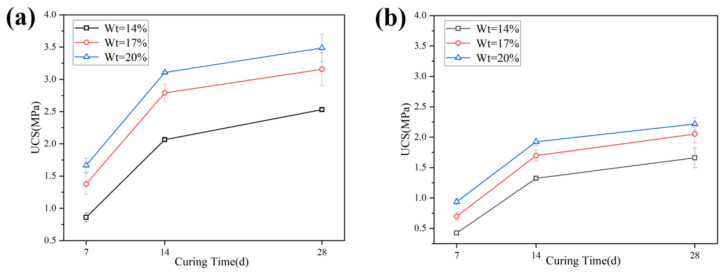

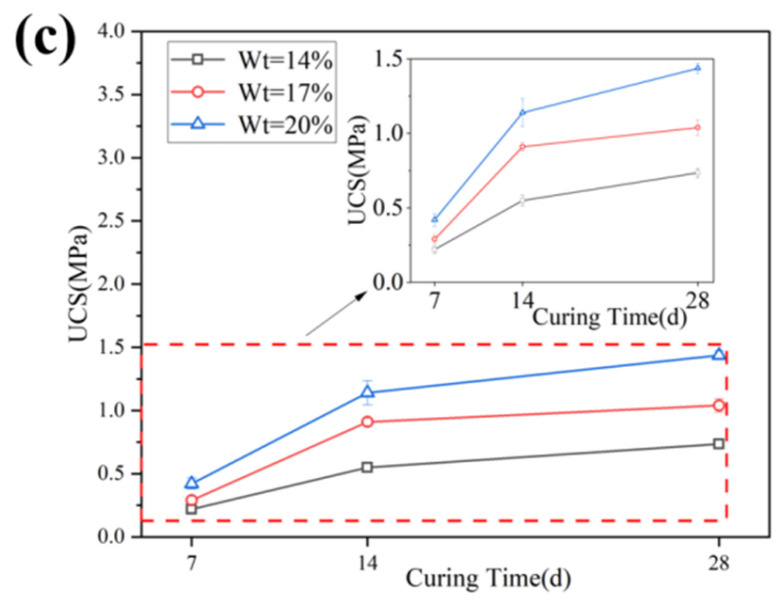
The influence of UCS on curing time: (**a**) the UCS of solidified soil Ⅰ at different contents of solidifying agent; (**b**) the UCS of solidified soil Ⅱ at different contents of solidifying agent; (**c**) the UCS of solidified soil Ⅲ at different contents of solidifying agent.

Figure 4 (Wt represents the content of the solidifying agent) shows the variation in the UCS of stabilized soil with curing time. The UCS increases more rapidly between 7–14 days, and at 14 days, the strength can reach 80–90% of that at 28 days. Both stabilized soil Ⅰ and Ⅱ show similar regular patterns. When the content of the solidifying agent is 14%, 17%, and 20%, the growth rates of the UCS between 14 and 28 days are 15.58%, 11.71%; 10.89%; and 20.48%, 17.07%, and 13.51%, respectively. It should be noted that the UCS of solidified soil Ⅲ was significantly lower than that of solidified soils Ⅰ and Ⅱ, especially when the content of the solidifying agent was 14%. The UCS of solidified soil Ⅲ at 7 days, 14 days, and 28 days were 0.22 MPa, 0.55 MPa, and 0.73 MPa, respectively. The main reason for this may be that soil Ⅲ has a high water content, which weakens the solidifying effect of the solid-waste-based binder. Simultaneously, the humic acid of the organic matter in soil Ⅲ can also destroy the hydration structure, resulting in lower strength [36,37]. Despite that, the UCS of solidified soils Ⅰ, Ⅱ, and Ⅲ after 28 days can both satisfy the technical requirements for the cement–soil mixing pile (>0.8 MPa, YBJ 225-91 [38], Chinese standard) when the solidifying agent content is 17% (corresponding to 60 kg/m of the cement–soil mixing pile).

#### 3.1.2. Stress–Strain Curves

The stress–strain curve can be divided into three stages [39]: (1) the linear elastic stage, during which the curve is approximately a straight line and the solidified soil undergoes elastic deformation; (2) the plastic deformation stage, in which the axial stress shows non-linear growth with axial strain, a slip occurs between the soil particles in the solidified soil, and small cracks appear on the surface of the solidified soil; (3) the failure stage, in which, as the axial strain increases, the axial stress begins to drop sharply and small cracks form inside the soil specimen leading to penetrative cracking and failure.

**Figure 5:**
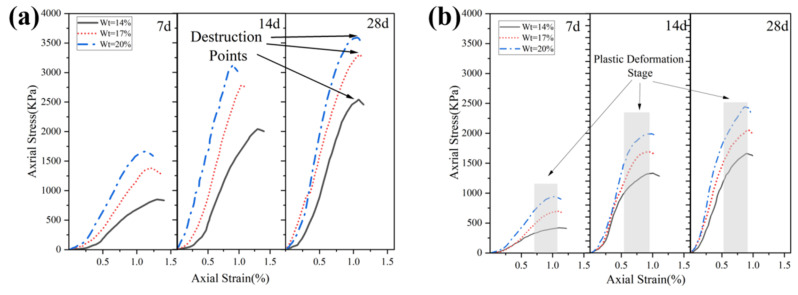

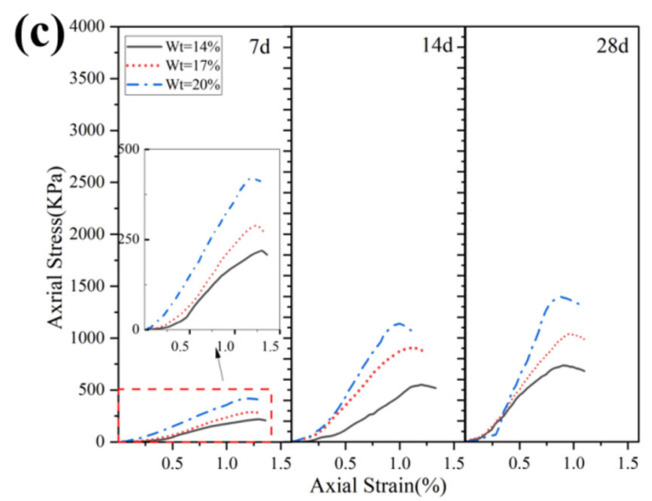
Stress–strain curves of solidified soil: (**a**) stress–strain curves of solidified soil Ⅰ at different contents of solidifying agent and curing times; (**b**) stress–strain curves of solidified soil Ⅱ at different contents of solidifying agent and curing times; (**c**) stress–strain curves of solidified soil Ⅲ at different contents of solidifying agent and curing times.

Figure 5 shows the stress–strain curves of solidified soils Ⅰ, Ⅱ, and Ⅲ. The axial failure strain of the solidified soil decreases as the solidifying agent content increases. As shown in Figure 5a, the elastic stage of solidified soil Ⅰ was prolonged, the peak stress increased, and the axial failure strain decreased from 1.0% to 0.75% (28 d) as the content of the solidifying agent increased from 14% to 20%. After the peak strength was reached, the strength of the solidified soil decreased rapidly, indicating brittle failure.

In Figure 5b, solidified soil Ⅱ (with a higher clay content, as shown in Table 2) shows a more pronounced plastic stage after the elastic stage, which was measured to be 0.74–1.08%, 0.59–0.95%, and 0.53–0.89% at 7 d, 14 d, and 28 d, respectively. The main reasons for this are that the cohesive force between the clay particles is weak, more stabilizers are needed to bond the particles together [40], and the higher clay content results in a lower failure stress and a more pronounced plastic stage.

The axial failure strain range of solidified soil Ⅲ was between 1.17% and 1.29% at 7 days of curing time (Figure 5c) and did not change significantly with increasing solidifying agent content, indicating that the strength of the solidified soil had not been fully developed during the 7 days of curing time and that the strength was growing slowly. The axial failure strain decreased to 1.0–1.19% and 0.87–0.96% when the curing time was 14 and 28 days, respectively. Compared to the axial failure strain of the early (7 d) solidified soil, the brittleness and strength of the solidified soil increased significantly between 14 and 28 days.

### 3.2. Analysis of Mineralogical and Microstructural Test Results

#### 3.2.1. XRD Tests

**Figure 6:**
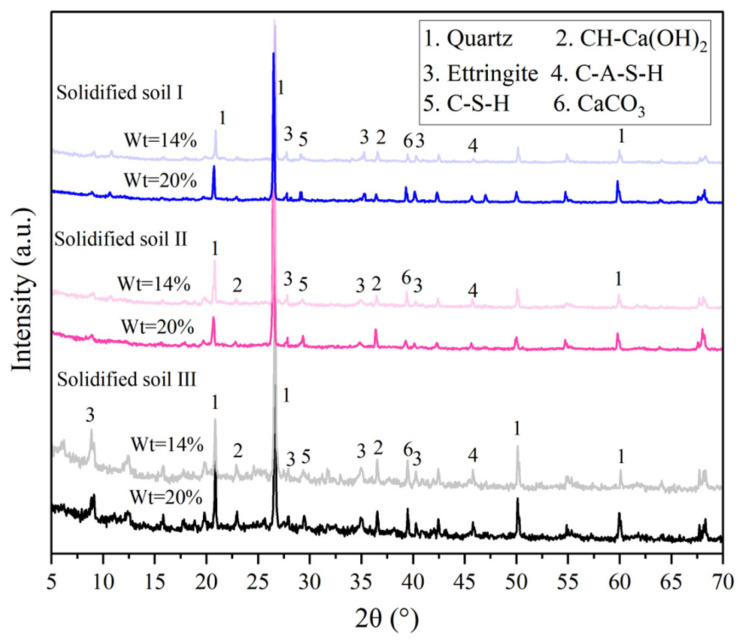
XRD tests of solidified soil. Phases identified: Quartz, PDF# 46-1045; CH, PDF# 04-0733; ettringite, PDF# 41-1451; C-A-S-H, PDF# 32-0151; C-S-H, PDF# 14-0035; CaCO_3_, PDF# 05-0586.

XRD tests were conducted on the solidified soil samples with solidifying agent contents of 14% and 20%. The XRD spectrums of the samples for 28 days of curing are shown in Figure 6. The results indicate that the main mineral phases in the solidified soil are CH (Ca(OH)_2_), ettringite, C-S-H, and C-A-S-H. By comparing the solidified soil samples with the same curing time, it was found that the hydrated products such as C-S-H, CH, and ettringite had stronger diffraction peak intensity at wt = 20%, indicating a higher proportion of hydration products that can bind soil particles and form a denser structure [25].

#### 3.2.2. SEM Tests

**Figure 7:**
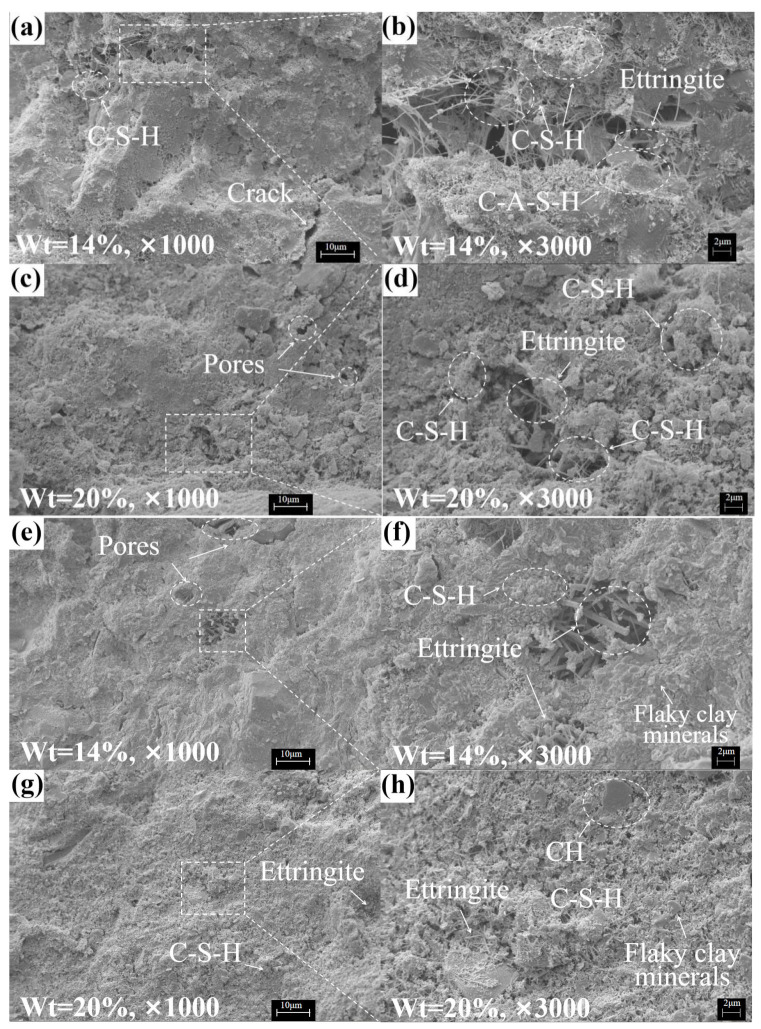

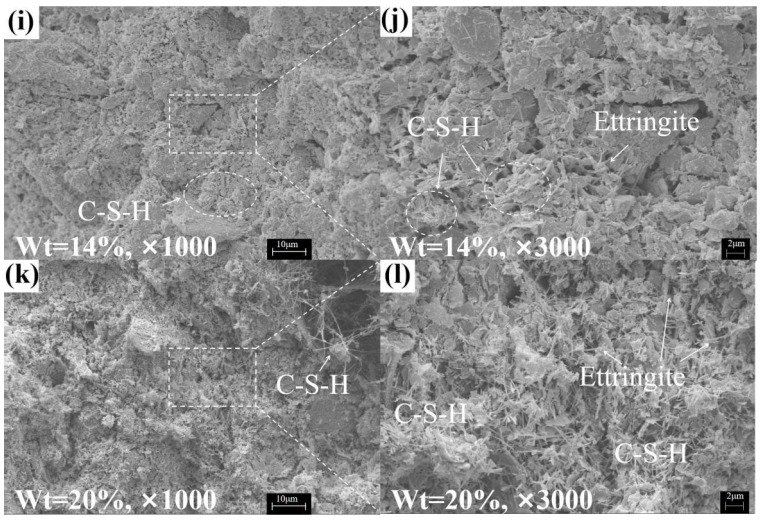
Microscopic morphology of different solidified soil samples. Solidified soil Ⅰ: (**a**,**b**) Wt = 14%; (**c**,**d**) Wt = 20%. Solidified soil Ⅱ: (**e**,**f**) Wt = 14%; (**g**,**h**) Wt = 20%. Solidified soil Ⅲ: (**i**,**j**) Wt = 14%; (**k**,**l**) Wt = 20%.

The micromorphologies of solidified soil (at 28 days) with different solidifying agent contents are shown in Figure 7. According to the XRD analysis and morphological characteristics [17,41,42], it was observed that a large amount of C-S-H and ettringite were generated in each sample, transforming the soil particle structure to a flocculent type with a plate-like or agglomerated morphology composed of soil–gel clusters (Figure 7a,h,j). As the content of the solidifying agent increased, the connection between the soil particles became more compact and dense, and cracks and pores tended to change from large to tiny. Additionally, in solidified soil Ⅱ (Figure 7h), a relatively dense matrix was found to be formed by a mixture of CH crystals and amorphous C-S-H. Figure 7b shows that the mesh-like C-S-H and plate-like C-A-S-H generated in solidified soil Ⅰ were staggered and stacked into a block-like structure, enhancing the adhesive force between soil particles. With the increasing content of the solidifying agent, the gap between the gel clusters decreases, and the gap between clusters was filled with ettringite, leading to an increase in the compaction and strength of solidified soil Ⅰ. Plate-like clay minerals were wrapped by a large amount of C-S-H (Figure 7j), and the ettringite generated was filled into the pores (Figure 7e,f,k), leading to an increase in the compactness of the solidified soil via cementation and pore filling. In addition, the plate-like clay minerals significantly increased the plasticity of the solidified soil [43], which is consistent with the analysis of the stress–strain curves.

#### 3.2.3. TGA Tests

**Figure 8:**
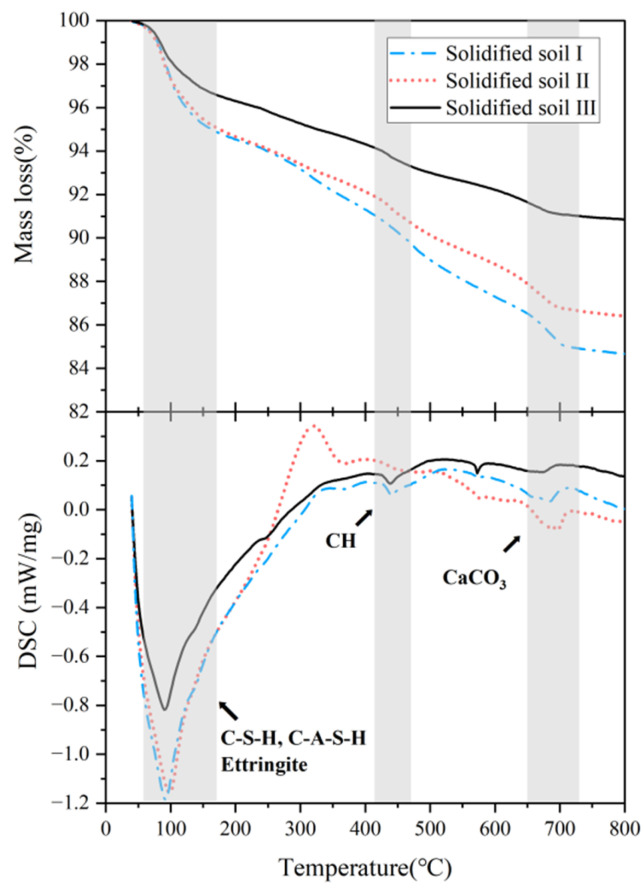
TGA test results of solidified soil.

Figure 8 shows the TGA&DSC curve of the solidified soil for 28 days with a solidifying agent content of 20%. By comparing these curves, it was found that the mass loss located at approximately 100 °C was caused by the dehydration reactions of hydration products C-S-H, C-A-S-H, and ettringite [25,44]; the mass loss at 400–500 °C was caused by the dehydroxylation of calcium hydroxide (CH) [45,46,47]; and the main mass loss at 650–750 °C was caused by the decarbonation (or carbonate decomposition) of calcium carbonate (CaCO_3_) [13,48]. In addition, the first endothermic peak of solidified soil Ⅲ in the curve was relatively lower than that of solidified soil Ⅰ and Ⅱ, indicating that the amount of hydration products, such as C-S-H and C-A-S-H, generated in solidified soil Ⅲ was less than that in solidified soil Ⅰ and Ⅱ. These hydration products are the main components contributing to the strength of solidified soil [25]. A higher content of C-S-H and AFt results in higher strength. Therefore, the result of TGA&DSC is consistent with the results of the UCS tests.

## 4. Analysis of Solidification Mechanism

After blending the solidifying agent with the soft soil, a series of physical and chemical reactions take place between the solid-waste-based binder and the soil, including agglomeration, ion exchange, hydration, and volcanic ash reactions. The Ca^2+^ dissolved from CaO and Ca(OH)_2_ is enriched on the surface of soil particles, replacing the Na^+^ and K^+^ ions adsorbed on the surface of soil particles, reducing the thickness of the electrical double layer, reducing the repulsion between soil particles, and promoting the agglomeration and flocculation of soil particles [49]. Calcium hydroxide is adsorbed on the surface particles of the waste ceramic powder and reacts with its active components to produce gelation products such as C-S-H and C-A-H via the volcanic ash reaction. The Si-O-Si and Al-O-Al bonds in slag and the Si-O-Si bonds in steel slag are broken when the pH value of the binder slurry exceeds 13.15 [50]. Al_2_O_3_ and SiO_2_ dissolve from the slag glass phase in the form of [SiO_4_]^4−^ and [AlO_4_]^5−^, as shown in Equations (5) and (6), and react with Ca^+^ in the system to form C-S-H and C-A-S-H gels, as shown in Equations (7) and (8). These hydration products are the main contributors to the strength of the solidified soil.

(5) $$-Si-O-Si\overset{OH^{-}}{\to }{\left[SiO_{4}\right]}^{4-}$$

(6) $$-\mathrm{Al}-O-Al\overset{OH^{-}}{\to }{\left[AlO_{4}\right]}^{5-}$$

(7) $$Ca^{2+}+{\left[SiO_{4}\right]}^{4-}+(n-1)H_{2}O\overset{OH^{-}}{\to }CaO\cdot SiO_{2}\cdot nH_{2}O(C-S-H)$$

(8) $$Ca^{2+}+{\left[SiO_{4}\right]}^{4-}+{\left[AlO_{4}\right]}^{5-}+nH_{2}O\overset{OH^{-}}{\to }3CaO\cdot Al_{2}O_{3}\cdot 2SiO_{2}\cdot nH_{2}O(C-A-S-H)$$

[AlO_4_]^5−^ can react rapidly with SO_4_^2−^ in desulfurized gypsum to generate expansive ettringite, which can effectively fill the blocky pores in the solidified soil and increase its density, as shown in Equation (9) below. Soft soils usually have high porosity and relatively large pores. For example, the pore size distribution of cement stabilized soil is between 300–1000 nm [51]. Ettringite can increase the volume of the solid phase by 120%, effectively fill the blocky pores in the solidified soil, and increase its density.

(9) $$Ca^{2+}+{\left[AlO_{4}\right]}^{5-}+SO_{4}^{2-}\overset{OH^{-}}{\to }3CaO\cdot Al_{2}O_{3}\cdot 3CaSO_{4}\cdot 32H_{2}O$$

## 5. Conclusions

The macroscopic mechanical properties of the soft soil solidified by the solid-waste-based binder were studied by UCS tests. The microstructure of the solidified soil was studied by XRD, SEM, and TG tests, and its solidification mechanism was revealed. The main conclusions were as follows:

(1) Based on three ratios and the strength activity index originally developed for the cement clinker, a unified method for solidifying soft soil using various types of solid waste has been achieved.
(2) The solid-waste-based binder effectively improved the strength of three types of soft soils (silty sand, silty clay, and organic clay). The 14-day UCS strength of the solidified soil reached 80–90% on day 28, and the UCS of both types of solidified soil with a solidifying agent content of 17% was 0.8 MPa or more at 28 days.
(3) With the increase in solidifying agent content and curing time, the brittleness of the solidified soil increased, and the solidified soil with the higher clay content showed greater plasticity.
(4) With the increase in solidifying agent content, the pores in the solidified soil evolve from large to tiny, and the pores are filled with a large amount of gel substance and needle-like ettringite crystals. The main hydration products or volcanic ash products in soft soils solidified by the solid-waste-based binder include CH, C-S-H, and ettringite. These products are the main contributors to the strength of solidified soil.

## Figures and Tables

**Table 1:** A framework for multi-source solid-waste-based soft soil solidification materials.

Components of Solid Waste	A	B	C	D	SAI
CaO	A1	B1	C1	D1	η_A_
SiO_2_	A2	B2	C2	D2	η_B_
Al_2_O_3_	A3	B3	C3	D3	η_C_
Fe_2_O_3_	A4	B4	C4	D4	η_D_
(CaO)act	A1 × η_A_	B1 × η_B_	C1 × η_C_	D1 × η_D_	/
(SiO_2_)act	A2 × η_A_	B2 × η_B_	C2 × η_C_	D2 × η_D_	/
(Al_2_O_3_)act	A3 × η_A_	B3 × η_B_	C3 × η_C_	D3 × η_D_	/
(Fe_2_O_3_)act	A4 × η_A_	B4 × η_B_	C4 × η_C_	D4 × η_D_	/
KH	$\frac{\left(CaO\right)\mathrm{act}-1.65\left(Al_{2}O_{3}\right)\mathrm{act}-0.35(Fe_{2}O_{3})act}{2.8(SiO_{2})act},\;0.66\leq KH\leq 1.0$				
SM	$\frac{({\mathrm{SiO}}_{2})\mathrm{act}}{\left({\mathrm{Al}}_{2}O_{3}\right)\mathrm{act}+({\mathrm{Fe}}_{2}O_{3})\mathrm{act}},\;1.7\leq SM\leq 2.7$				
IM	$\frac{\left(Al_{2}O_{3}\right)\mathrm{act}}{(Fe_{2}O_{3})act},0.9\leq IM\leq 1.7$				
Solidifying agent	Ⅰ%A + Ⅱ%B + Ⅲ%C + Ⅳ%D + ······				

**Table 2:** Geotechnical properties of the studied soil.

Test Soil	Ⅰ	Ⅱ	Ⅲ
Natural water content/%	32.32	36.05	50.81
Wet density, g/cm^3^	1.87	1.66	1.55
Liquid limit/%	29.79	35.47	64.93
Plastic limit/%	12.54	22.94	29.57
Organic matter content/%	/	/	5.31
Sand fraction (2–0.074 mm, %)	66.65	3.17	11.20
Silt fraction (0.074–0.002 mm, %)	30.03	49.42	74.55
Clay fraction (<0.002 mm, %)	3.32	47.41	14.25
Unified Soil Classification System classification	Silty sand (SM)	Silty clay (CL)	Organic clay (OH)

**Table 3:** Main components (wt%) of solid waste and lime used in this work.

Components	Ground Granulated Blast Furnace Slag	Steel Slag	Waste Ceramic Powder	Red Mud	Desulfurization Gypsum	Lime
CaO	39.78	39.52	1.05	26.36	33.12	97.88
SiO_2_	32.34	13.06	70.64	25.79	1.16	/
Al_2_O_3_	16.24	3.37	16.12	11.06	1.67	/
Fe_2_O_3_	0.51	26.47	0.75	7.39	0.38	/
MgO	6.17	2.33	1.49	1.01	/	/
SO_3_	1.64	0.22	0.03	1.17	40.84	/
Na_2_O	0.32	0.93	3.35	1.11	/	/
Loss on ignition	0.32	0.68	2.63	11.72	14.2	/

**Table 4:** The strength activity index of solid waste.

Solid Waste	Ground Granulated Blast Furnace Slag	Steel Slag	Waste Ceramic Powder	Red Mud
SAI	1.02	0.70	0.89	0.58

## Data Availability

All data, models, and code generated or used during the study appear in the submitted article.
